# Health-related social media use and COVID-19 anxiety in adolescence: health anxiety as covariate and moderator

**DOI:** 10.3389/fpsyg.2023.1079730

**Published:** 2023-05-02

**Authors:** Adela Lokajova, David Smahel, Nikol Kvardova

**Affiliations:** Interdisciplinary Research Team on Internet and Society, Faculty of Social Studies, Masaryk University, Brno, Czechia

**Keywords:** digital health, adolescence, health anxiety, COVID-19 anxiety, COVID-19 experience

## Abstract

**Background:**

Adolescents can benefit from engagement with health-related content on social media (e.g., viewing, commenting, or sharing content related to diseases, prevention, or healthy lifestyle). Nevertheless, such content may be distressing or exaggerated and present a challenge to mental well-being, especially during the COVID-19 pandemic. Rumination about such content may lead to COVID-19 anxiety. Yet, the individual factors that would explain the association between health-related social media use (SMU) and COVID-19 anxiety are understudied.

**Objective:**

In the current study, we aimed to fill the gap by investigating the association between health-related social media use (SMU) and COVID-19 anxiety in light of several individual factors: health anxiety, eHealth literacy, and mild and severe experience with COVID-19 infection. We (1) studied the relationship between individual factors and health-related SMU, (2) tested health anxiety as a moderator in the association between health-related SMU and COVID-19 anxiety, and (3) explored a direct effect of experience with COVID-19 on COVID-19 anxiety.

**Methods:**

Using structural equation modeling, we analyzed cross-sectional data from a representative sample of 2,500 Czech adolescents aged 11–16, 50% girls. Sociodemographic measures, health-related SMU, COVIDCOVID-19 anxiety, health anxiety, eHealth literacy, and mild and severe experience with COVID-19 infection were assessed with an anonymous online survey. The data were collected in June 2021.

**Results:**

We conducted a path analysis to test the main relationships and an additional simple-slopes analysis to explore the moderating effect of health anxiety. Higher health anxiety and eHealth literacy were associated with increased health-related SMU. The effect of experience with COVID-19 infection on both COVID-19 anxiety and health-related SMU was negligible. Health-related SMU and COVID-19 anxiety were positively associated, however, only for adolescents high in health anxiety. For other adolescents, the two variables were unrelated.

**Conclusion:**

Our findings show that adolescents with higher health anxiety and eHealth literacy engage in health-related SMU more intensively. Furthermore, for adolescents high in health anxiety, the frequency of health-related SMU is associated with the risk of COVID-19 anxiety. This is likely due to differences in media use. Adolescents with high health anxiety may use social media for content that is more likely to lead to COVID-19 anxiety compared to other adolescents. We recommend focusing on the identification of such content, which may lead to more precise recommendations regarding health-related SMU compared to cut-back on the frequency of overall SMU.

## 1. Introduction

The COVID-19 pandemic has presented many sources of distress for adolescents, such as isolation, learning online, or worrying about health (Hörbo et al., 2021), and has impacted their mental health (Fruehwirth et al., 2021). The prevalence of clinically elevated anxiety has risen from 11.6% (pre-pandemic state) to 20.5% in 2021 (Racine et al., 2021), and sub-clinical anxiety can be expected to be more frequent. While the increase can be partially attributed to losing access to intervention, such as school psychologists (Racine et al., 2021), the pandemic itself can explain a significant ratio.

Apparently, most people were resilient to the pandemic situation, adapted to it, or could even benefit from it in terms of mental health (Shevlin et al., 2021). However, a certain group of people, commonly those already in mental health treatment, appeared to be more vulnerable to the situation and were developing mental health problems as the pandemic progressed (Shevlin et al., 2021). Continuous exposure to stressors may lead to COVID-19 anxiety, i.e., acute anxiety linked to the COVID-19 pandemic (Fu et al., 2021) and its reflection in the media (Lee, 2020a). According to Lee (2020a), COVID-19 anxiety is triggered by thoughts or information about the pandemic. COVID-19 anxiety may be a gateway to long-term problems (Lee, 2020b).

Social media present an essential platform for adolescents, and their importance has grown due to social distancing (Hussong et al., 2021). However, following health-related (and pandemic-related) content on social media may also contribute to COVID-19 anxiety (Mertens et al., 2020) show that following health-related (and pandemic-related) content on social media may contribute to COVID-19 anxiety. Nonetheless, research on the relationship between health-related social media use (SMU) and anxiety, specifically COVID-19 anxiety, remains scarce (Keles et al., 2020; Valkenburg et al., 2022), especially with regard to adolescents. We aim to study this relationship along with variables that may influence it. Moreover, most of the studies come from early 2020 when, compared to the present, exposure to COVID-19 was uncommon and preventive measures had not yet been implemented. We aim to add to the literature by presenting data from a more recent wave of the COVID-19 pandemic.

In line with our aims, we frame this study by the Differential Susceptibility to Media Effects Model (DSMM; Valkenburg and Peter, 2013). The DSMM proposes that the relationship between media use (e.g., health-related SMU) and its effects (e.g., COVID-19 anxiety) is affected by, i.e., media and non-media variables that frame individual behavior. This mechanism is described in four propositions: (1): media use depends on three types of differential susceptibility variables: developmental, dispositional, and social; (2) the relationship between media use and media effects is indirect, and it is mediated by short-term response states, which are cognitive, emotional, or excitative; (3) the differential susceptibility variables play two roles in the model. First, they predict the media use, and second, they moderate the relationship between the media use and the response states; and (4) media effects are transactional and can impact further media use, response states, and differential susceptibility variables. The third proposition may be viewed as the most innovative one as it implies that a single variable appears twice in a single model in two different roles. Yet, it is imaginable that an individual variable such as trait or skill affects not only the adoption of a certain media use but also the process and its outcome. Previous studies have shown that this proposition is viable and inserting one variable both as a predictor and a moderator can contribute to better understanding of the studied phenomena (Landrum et al., 2019; Kvardova et al., 2021). Therefore, we will utilize this proposition.

This study adopts an adaptation of the DSMM by Dedkova et al. (2022), tailored to information and communication technologies (ICT) and their relationship with well-being. The terminology in Dedkova et al. (2022) is more suitable for the current study, and that offers additional insights into media use. The differential susceptibility variables are reorganized into individual-, social-, and country-level ones. The conceptualization of media use is more developed and encourages the definition of ICT use more precisely (e.g., passive/active, excessive/balanced). Finally, response states and media effects are replaced by short-term (e.g., mood alterations) and long-term (e.g., loneliness) well-being in psychological, social, and physical dimensions (Dedkova et al., 2022).

COVID-19 anxiety appears to fit the short-term category (compared to, for example, general anxiety), and it will be handled as such. In the conceptualization by Lee (2020a), COVID-19 anxiety means acute somatic symptoms of anxiety (e.g., sleep disturbances, rumination, or nausea) related to engaging with information about the COVID-19 pandemic during the past 2 weeks.

Social media use may present a source of such information. Despite being an essential tool for communicating, obtaining news, and sharing knowledge during pandemics (Bendau et al., 2021), social media may present a kind of online activity leading to distress. During the pandemic, the news was often disturbing. Even following accurate sources of information could decrease well-being (Tsamakis et al., 2021), not to mention more exaggerated, underestimated, and misleading content (Bendau et al., 2021). COVID-19 anxiety seems to be related to engaging with information about the disease or the pandemic itself and other health-related issues (Shabahang et al., 2020). Also, the frequency of engaging with media, rather than the source, seems to be an important factor for COVID-19 anxiety (Bendau et al., 2021).

The passivity or activity of SMU appears to be another possible factor. Escobar-Viera et al. (2018) perceive passive SMU as consuming content without any interaction (i.e., reading or viewing others’ content). The active SMU includes creating posts and interacting with the content (i.e., reposting, liking, or commenting; Escobar-Viera et al., 2018). Passive health-related SMU seems to follow the outlined trend; that is, more frequent SMU is related to various kinds of adverse outcomes, such as depression or anxiety (Thorisdottir et al., 2019; Valkenburg et al., 2022). Therefore, the following is expected:

*H1a*: Passive health-related SMU is positively related to COVID-19 anxiety.

Active health-related SMU seems to work in a less stable way. Soroya et al. (2021) argue that, unlike passive health-related SMU, active health-related SMU does not cause information overload that leads to anxiety. Others argue that active health-related SMU may still be related to increased anxiety (Valkenburg et al., 2022), although the effect would disappear after controlling for other variables, such as peer support (Thorisdottir et al., 2019). Altogether, the findings are contradictory and difficult to generalize (Keles et al., 2020), and there is no study focused on active health-related SMU and COVID-19 anxiety specifically. Therefore, we study the effect of active health-related SMU separately for exploratory reasons. The contradiction in the results does not allow us to perceive any direction of effect of active health-related SMU on COVID-19 as more likely than the others. Therefore, we decided to formulate a “pseudo-null” hypothesis and to presume that active health-related SMU does not differ from the passive and overall health-related SMU:

*H1b*: Active health-related SMU is positively related to COVID-19 anxiety.

As outlined by the DSMM, the proposed relationship between health-related SMU and COVID-19 anxiety does not have to be the same for everyone. Individual variables may determine whether and with what result adolescents use health-related media. The current study focuses on three such variables: health anxiety, eHealth literacy, and experience with COVID-19 infection.

First, users may differ in their motivation associated with health-related SMU. Health anxiety presents an individual trait that may drive the process of health-related SMU. It represents body-related distress and rumination manifested through unreasonable fear of serious diseases, overestimation of their occurrence, and excessive attention to bodily symptoms (Rachman, 2012). People with health anxiety tend to engage in behaviors they believe would reduce the anxiety but typically only lead to its increase (Rimvall et al., 2021). Health-related media use presents such behavior. Health anxiety often leads to increased health-related SMU, which is supposed to comfort health-related worries (Brown et al., 2020). This has been supported for various kinds and platforms of health-related media use, like engaging with patient fora (Baumgartner and Hartmann, 2011), seeking information about diseases (te Poel et al., 2016), and employing health-promoting activities (Singh and Brown, 2014). Health anxiety impacts both passive (e.g., reading articles) and active (e.g., posting to a forum) behaviors (Baumgartner and Hartmann, 2011; te Poel et al., 2016), which in turn may impact COVID-19 anxiety. However, to the best of our knowledge, this effect has never been explicitly tested for SMU. We hypothesize:

*H2a*: Health anxiety is positively related to passive health-related SMU.

*H2b*: Health anxiety is positively related to active health-related SMU.

Health anxiety seems to be further involved in the process of health-related SMU. For people high in health anxiety, health-related internet use leads to increased distress or anxiety compared to less health-anxious users (Baumgartner and Hartmann, 2011), and if not, it often encourages another potentially more distressful search (Starcevic and Berle, 2013). People higher in health anxiety also engage with health-related information more intensively, which makes them prone to distressful experiences, such as escalation of the query and learning about a new distressing condition (Singh and Brown, 2016). People with higher health anxiety are also more likely to perceive information, even unverified, as more serious and are, therefore, more likely to share it (Oh and Lee, 2019). We expect that such an effect may be present not only between health-related SMU and unspecified distress or anxiety and that it may also strengthen the effect on short-term COVID-19 anxiety:

*H3a*: The relationship between passive health-related SMU and COVID-19 anxiety is stronger for adolescents with higher health anxiety.

*H3b*: The relationship between active health-related SMU and COVID-19 anxiety is stronger for adolescents with higher health anxiety.

Second, in order to engage with health-related social media content, one needs to possess a certain level of skills related to search and evaluation. eHealth literacy represents skills (i.e., digital skills and health literacy) needed to navigate through health-related information online (Norman and Skinner, 2006). People who are more eHealth-literate tend to use the internet for health-related purposes more often (Wong and Cheung, 2019) and benefit more from the information (Li et al., 2014). For adolescents, subjective eHealth literacy is often unrelated to the objective level of this skill (Maitz et al., 2020). Nonetheless, the subjective level of the trait remains relevant for the frequency of health-related SMU and may increase the user’s confidence and enable health-related internet use (Chang et al., 2015; Wong and Cheung, 2019). Therefore, we believe that subjective eHealth literacy presents a valid variable in health-related SMU, and we hypothesize:

*H4a*: eHealth literacy is positively related to passive health-related SMU.

*H4b*: eHealth literacy is positively related to active health-related SMU.

And third, during the COVID-19 pandemic, one’s levels of media use (Houston et al., 2018) and COVID-19 anxiety are likely impacted by one’s experience with the pandemic. When considering health-related SMU, it seems reasonable to assess an experience linked to COVID-19 as a disease rather than, for example, online schooling or the economic situation. Authors have typically assessed one’s experience with COVID-19 based either on proximity (Attema et al., 2021) or severity (Fruehwirth et al., 2021). The latter approach seems to reflect the position somewhat better because experiences with mild and severe COVID-19 are likely non-additive. The impact of experience with a mild COVID-19 infection on anxiety is inconsistent (Cao et al., 2020; Fruehwirth et al., 2021). Likely, the experience may present a source of distress for some, while for others, an encounter with its mild form can actually bring relief. The effect may also evolve over time. The effect on anxiety can be found in studies from early 2020 (Cao et al., 2020) when personal experience with COVID-19 was uncommon. Later studies fail to confirm such results (Fruehwirth et al., 2021). Contrarily, experience with hospitalization or death because of COVID-19 presents a consistent source of distress and anxiety (Milman et al., 2020). Therefore, we consider both kinds of experiences separately.

The impact of COVID-19 experience on health-related SMU remains understudied. The rates of health-related internet use grow with the rise of COVID-19 cases (Kushwaha et al., 2021); however, it is unclear whether this is related to personal COVID-19 experience. While experience with a relative being hospitalized seems to increase the amount of online health information seeking (Chen et al., 2021), long-term patterns or the use in milder conditions are unknown. However, guided by Houston et al. (2018) and the aforementioned papers, we hypothesize:

*H5a,b*: The experience with mild COVID-19 is related to passive (H5a) and active (H5b) health-related SMU.

*H5c,d*: The experience with severe COVID-19 is related to passive (H5c) and active (H5d) health-related SMU.

The link between COVID-19 experience and COVID-19 (and general) anxiety has been studied before. In Milman et al. (2020), encountering COVID-19 was related to increased COVID-19 anxiety in adults, while variables, such as childcare loss or unemployment due to the pandemic, had no effect. Others report similar results for college students (Cao et al., 2020). However, Fruehwirth et al. (2021) did not find any relationship between first-year college students’ COVID-19 experience and anxiety. Noteworthily, the data in Fruehwirth et al. (2021) were collected during a calmer time in the summer of 2020. This would suggest that the relationship between COVID-19 experience and COVID-19 anxiety may evolve during the pandemic. We aim to explore a more recent state of this relationship, and we hypothesize:

*H6a*: Experience with mild COVID-19 is related to COVID-19 anxiety.

*H6b*: Experience with severe COVID-19 is related to COVID-19 anxiety.

Additionally, we controlled the effect of age and the gender of the participants on health-related SMU. Girls have been shown to use the internet for health purposes more often than boys. Similarly, the frequency rises with age as younger adolescents still prefer consulting their parents (Park and Kwon, 2018). We also controlled the direct effect of gender on COVID-19 anxiety, which appears to be more common in girls (Akgül and Atalan Ergin, 2021). Conversely, age seems unrelated to COVID-19 anxiety, except for the most vulnerable population (Özdin and Bayrak Özdin, 2020).

To sum up, previous studies have shown that the pandemic may lead to acute COVID-19 anxiety. Nonetheless, factors associated with COVID-19 anxiety in adolescents remain understudied. We aim to bridge this gap and test how health-related SMU is associated with adolescent COVID-19 anxiety (H1).

**Figure 1:**
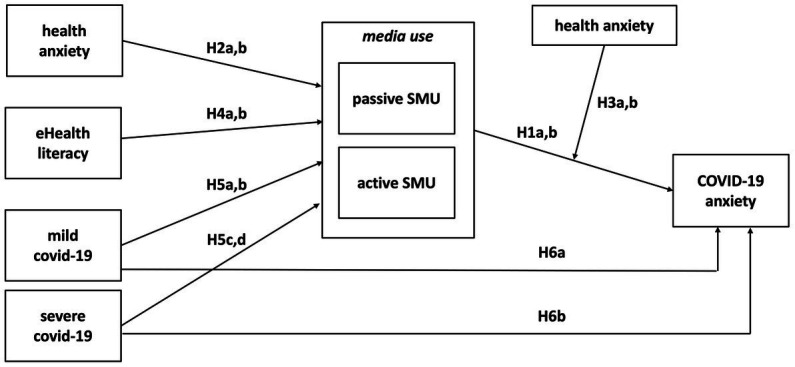
The conceptual model, with hypotheses.

In line with the DSMM, this relationship will be studied in the context of several individual variables (see Figure 1). We expect health anxiety to predict SMU (H2) and moderate its relationship with COVID-19 anxiety (H3). We test whether eHealth literacy is related to health-related SMU (H4). Based on Houston et al. (2018), we explore whether experience with mild and severe COVID-19 is associated with adolescent health-related SMU (H5).

Additionally, we test the direct association between experience with mild and severe COVID-19 and COVID-19 anxiety itself (H6), studied only for the older population and during the first months of the pandemic.

## 2. Materials and methods

### 2.1. Participants and procedures

We obtained data from 2,500 Czech adolescents (50% girls) aged 11 to 16 (*M* = 13.4, SD = 1.7) and their caregivers (63.6% female, *M_age_* = 42.8, SD = 7.0). The representation of individual age groups was nearly equal and ranged from 15.5% (16-year-olds) to 17.3% (12-year-olds). The data were collected by a market research agency in June 2021 via an online survey as part of the project Modeling the Future: Understanding the impact of technology on adolescents’ well-being. The agency recruited participants from their online panel and their children with quota sampling. Eligible were panelists who were caregivers to an adolescent aged 11 to 16 and shared the household with them. The education of the head of the family, municipality size, and region, according to NUTS 3, represented Czech households with children. For a more detailed description of the sample see Table 1. From an initial pool of 17,502 panel members, 6,818 opened the questionnaire and answered eligibility questions; a total of 3,396 were rejected as not eligible or because the quota was filled. Out of the households that completed the questionnaire, 624 were excluded, because their answers contained more than 10% missing data or were disqualified in data quality check. The respondents were assured anonymity and had the option to answer “I do not know” or “I do not want to answer” to any question. Parents were asked to grant their child privacy when filling out the questionnaire. Informed consent was given by the child and the caregiver. The survey was approved by the Ethical Committee of Masaryk University (Table 1).

**Table 1:** Respondent characteristics.

	*N*	%
Education (Head of the household)		
Unfinished primary	5	0.2
Primary	101	4.0
Secondary without GCSE	722	28.9
Secondary with GCSE	1,026	41.0
Tertiary	646	25.8
School type (adolescent)		
Primary school	1,883	75.3
Grammar school	273	10.9
Vocational school	307	12.3
Other type of regular school attendance	17	0.7
Home-schooled^a^	9	0.4
Other	11	0.4
Municipality size		
999 or less	338	13.5
1,000–9,999	778	31.1
10,000–49,000	565	22.6
50,000–99,999	254	10.2
100,000 and more	565	22.6

a Does not include temporary distant education during lockdowns.

### 2.2. Specifics related to COVID-19

The data were collected at the end of the third pandemic wave, which started at the end of January and culminated in the middle of March 2021. For the Czech Republic, it was the most severe pandemic wave to that point, with the capacities in hospitals reaching their limits. The restrictions were strict; from March to April, it was forbidden to leave the municipality. At the time of the data collection, the adolescents were newly attending offline classes after having been home-schooled for most of the school year, and restrictions were slowly being abandoned. The total rates of people infected with COVID-19 steadily decreased during the data collection and were at their lowest from 2021 (MZČR, 2022).

### 2.3. Measures

#### 2.3.1. COVID anxiety

Anxiety related to COVID-19 was measured with four items from the Coronavirus Anxiety Scale (CAS; Lee, 2020b), which represented acute bodily symptoms related to thinking about or being exposed to information about COVID-19 – dizziness, sleep disturbances, eating problems, and nausea – over the preceding 2 weeks. The response slightly differed from the original CAS and ranged from 1 *(never)* to 5 *(very often)*. However, both this study and Lee (2020b) use a scaling system based on the DSM-5’s cross-cutting symptom measure (American Psychiatric Association, 2013, pp. 734–735). The reliability of the scale was good, *ω* = 0.89 (*M* = 1.60, SD = 0.77), and the item-rest correlations were high (> 0.74).

#### 2.3.2. Health-related SMU

Health-related SMU was assessed with a modification to the scale by Escobar-Viera et al. (2018). We asked the following: “On the internet, one can find various contents (photos, videos, text) regarding health or illness, including COVID-19. How often do you engage in the following behaviors?” The passive behaviors include (1) reading articles or posts with such content, (2) reading discussions with such content, and (3) watching videos or viewing pictures with such content. The active behaviors were (4) replying to such content (e.g., liking, voting), (5) sharing others’ content (e.g., via Instagram stories, on Facebook), and (6) commenting on such content. The respondents answered on a seven-point scale from 1 *(never)* to 7 *(several times a day)*. The internal consistency for both the passive (*ω* = 0.89, *M* = 2.17, SD = 1.22) and active (*ω* = 0.89, *M* = 1.71, SD = 1.15) health-related SMU was good. The item-rest correlations were high for both the passive health-related SMU (*r* > 0.75) and active health-related SMU (*r* > 0.76).

#### 2.3.3. Health anxiety

Health anxiety was measured with four items from the Multidimensional Inventory of Hypochondriacal Traits (Longley et al., 2005). The adolescents indicated how true the following statements were for them: (1) I worry a lot about my health, (2) When I experience pain, I fear I might be ill, (3) Reading articles about disease makes me worry about my health, and (4) I am concerned with the possibility of being diagnosed with a serious disease. The respondents answered on a scale from 1 *(very untrue)* to 5 *(very true)*. The reliability of the scale was good, *ω* = 0.85 (*M* = 2.46, SD = 0.98), and the item-rest correlations were acceptably high (> 0.65).

#### 2.3.4. eHealth literacy

eHealth literacy was measured with five items from the eHealth Literacy Scale (eHEALS; Norman and Skinner, 2006), such as “I know what health resources are available on the internet” and “I have the skills I need to evaluate the health resources I find on the internet.” The respondents answered on a scale from 1 *(strongly disagree)* to 5 *(strongly agree)*. The reliability of the scale was good, ω = 0.88 (*M* = 3.34, SD = 0.88), and the item-rest correlations were acceptably high (> 0.69).

#### 2.3.5. Experience with mild and severe COVID-19

The adolescent’s experience with COVID-19 was reported by their caregiver with regard to the following situations: (1) This child knows somebody out of our family who has had COVID-19 without hospitalization, (2) This child knows somebody from our family who has had COVID-19 without hospitalization, and (3) This child knows somebody who underwent hospitalization or died because of COVID-19. The caregivers answered either 0 *(no)*, 1 *(yes)*, or 2 *(I do not know)*. The last option was considered a missing answer in the analysis. The experience with COVID-19 was handled as two variables that differed in severity: experience with mild COVID-19 (without hospitalization) and experience with severe COVID-19 (hospitalization or death), regardless of whether in the family or not.

#### 2.3.6. Demographics

Gender was determined by asking *“Are you.?”* with the responses (1) a girl or (2) a boy. Age was reported in years.

### 2.4. Statistical analysis

The data were analyzed using path analysis in Mplus (8th version, Muthén and Muthén, 2017). The variables were modeled as observed; those that consisted of multiple items were indicated by a mean. We standardized all variables except for gender (*M* = 0, SD = 1). The data were analyzed in two steps. First, we tested the model with the main effects (i.e., the effect of individual variables on health-related SMU, the effect of health-related SMU, and experience with COVID-19 on COVID-19 anxiety) and control variables (age and gender). Then we conducted two separate models with the addition of the moderation effects of health anxiety while controlling for the main effect of the moderating variable. We assessed RMSEA, SRMR, CFI, and TLI to evaluate the model to data fit. We followed the cut-off criteria suggested by Hu and Bentler (1999) of a good/acceptable fit of 0.95 for CFI and TLI, 0.06/0.08 for RMSEA, and 0.08 for SRMR. We used jamovi (version 1.6.23; The jamovi project, 2021) for the descriptive statistics of the participants (mean values for age, frequency of age groups) and the scales (mean values and standard deviations, skewness and kurtosis, internal consistency and item-rest correlations). We evaluated the internal consistency of the scales by item-rest correlations and McDonald’s omega. The omega coefficient should be higher than 0.70, ideally between 0.80 and 0.90 (Nunnaly and Bernstein, 1994), and the item-rest should be above 0.30 (Ferketich, 1991).

## 3. Results

### 3.1. Preliminarily analyses

The data were checked for assumptions, such as missing data, normal distributions, and homoscedasticity (Kline, 2012). For most variables, the amount of missing data did not exceed 1%. Three questions of the eHealth literacy scale had up to 2% missingness, and the variables that captured mild and severe COVID experience had 7 and 4% missing data, respectively. Regarding the missing data per participant, 85% of participants had no missing values in their responses, 13% had up to 9, and 2% had up to 48% of missing data. To examine the missing data patterns, we used Little’s MCAR test, which tests the null hypothesis that the data are missing completely at random (MCAR). Little’s test showed a statistically significant result, *χ*^2^ (1473) = 1,930.58, *p* < 0.001, suggesting that the data may not be MCAR. We used the Full Information Maximum Likelihood (FIML) approach to compensate for missing data since it assumes the data are missing at random (MAR) and does not require the MCAR assumption to be met (Dong and Peng, 2013). A Robust Maximum Likelihood Estimator was used because some of the variables were skewed. Initially, the model did not fit the data well (*RMSEA* = 0.38, *_90_CI* [0.36; 0.40], *CFI* = 0.60, *TLI* = −1.79, *SRMR* = 0.08). With regard to modification indices, we decided to allow for a correlation between active and passive health-related SMU. The two variables were strongly correlated (*r = 0*.69, *p* < 0.001), which is in line with previous evidence (Thorisdottir et al., 2019; Quiroz and Mickelson, 2021). After allowing this correlation, the fit improved significantly, and the model fitted the data adequately (*RMSEA* = 0.04, *_90_CI* [0.03; 0.05], *CFI* = 0.99, *TLI* = 0.97, *SRMR* = 0.02).

### 3.2. Descriptive statistics

Nearly three-quarters (71.7%) of the participants had experience with mild COVID-19, and 26.8% had experience with severe COVID-19. For other variables, see Table 2. Several of the variables were positively skewed. For COVID-19 anxiety, this reflects a general trend as the measures typically use response scales for the clinical population (Kubb and Foran, 2020). Both kinds of SMU were positively skewed. Still, over 2% of the participants reported engaging in both kinds of SMU on a daily basis.

**Table 2:** Descriptive statistics for continuous variables.

	*N*	Min	Max	*M*	SD	*Skew*.	*Kurt*.	*ω*
COVID-19 Anxiety	2,474	1	5	1.60	0.77	1.43	1.92	0.89
Passive health-related SMU	2,487	1	7	2.17	1.22	1	0.49	0.89
Active health-related SMU	2,476	1	7	1.71	1.15	1.82	1.82	0.89
Health Anxiety	2,461	1	5	2.46	0.98	0.41	0.41	0.85
eHealth Literacy	2,418	1	5	3.34	0.88	−0.56	−0.58	0.88

### 3.3. Results of the path analysis

**Figure 2:**
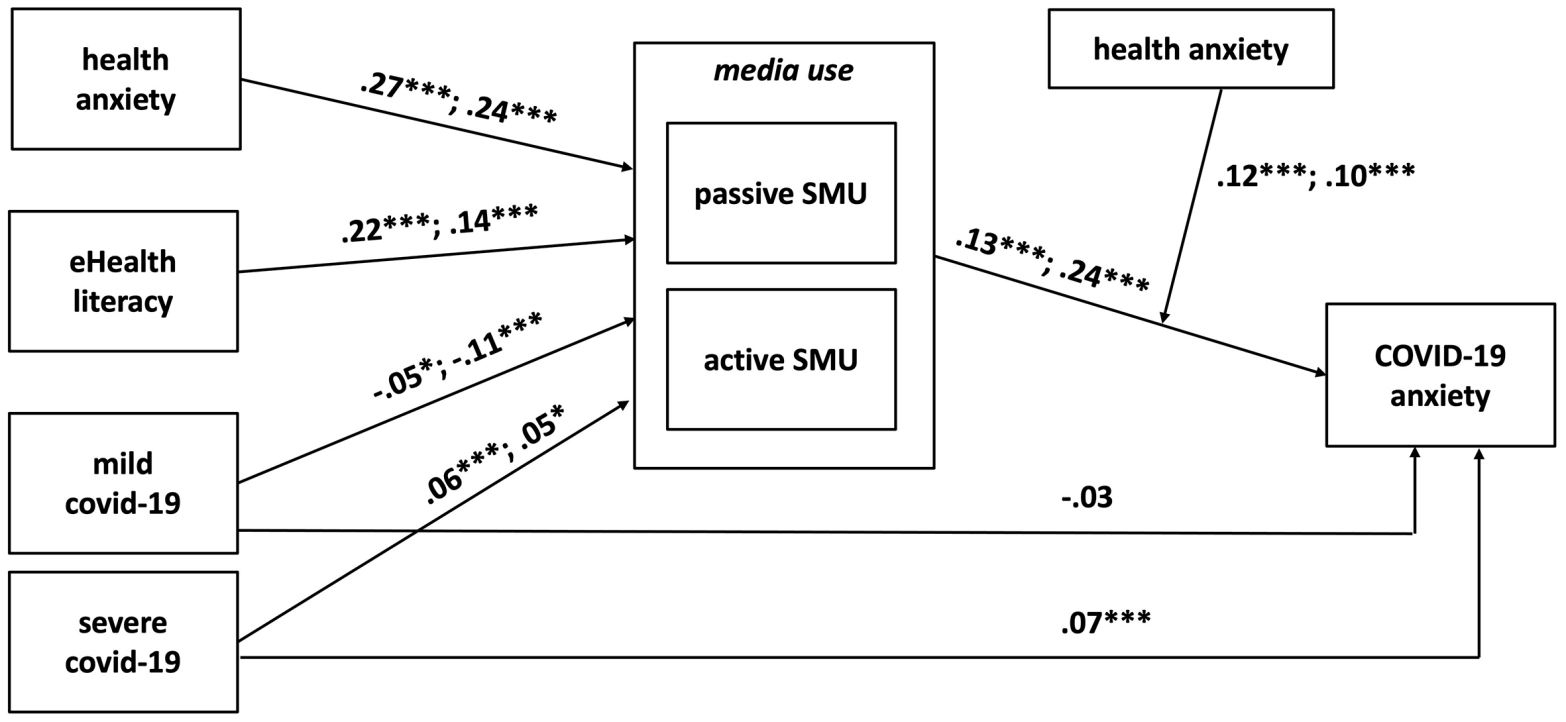
The conceptual model, with results. The first number on the description arrows is for passive health-related SMU and the second is for active health-related SMU. Age and gender were included as control variables but are not included for clarity reasons; their effects are reported in Table 2. *p < 0.05, **p < 0.01, ***p < 0.001.

Initially, the model did not fit the data well (*RMSEA* = 0.38, *_90_CI* [0.36; 0.40], *CFI* = 0.60, *TLI* = −1.79, *SRMR* = 0.08). With regard to modification indices, we decided to allow for a correlation between active and passive health-related SMU. The two variables were strongly correlated (*r* = 0.69, *p* < 0.001), which is in line with previous evidence (Thorisdottir et al., 2019; Quiroz and Mickelson, 2021). After allowing this correlation, the fit improved significantly, and the model fitted the data adequately (*RMSEA* = 0.04, *_90_CI* [0.03; 0.05], *CFI* = 0.99, *TLI* = 0.97, *SRMR* = 0.02). See the results in Table 3; Figure 2.

**Table 3:** The results for the main effects on health-related SMU.

	Passive health-related SMU				Active health-related SMU			
	*β*	_95_CI β	*b*	*p*	*β*	_95_CI β	*b*	*p*
Health anxiety	0.27	0.23; 0.30	0.33	<0.001	0.24	0.21; 0.28	0.29	<0.001
eHealth literacy	0.22	0.19; 0.25	0.31	<0.001	0.14	0.11; 0.18	0.19	<0.001
Experience with mild C-19	−0.05	−0.09; −0.01	−0.14	0.013	−0.11	−0.15; −0.06	−0.27	<0.001
Experience with severe C-19	0.06	0.02; 0.09	0.16	0.003	0.05	0.01; 0.09	0.13	<0.012
Gender	−0.01	−0.05; 0.03	−0.02	0.592	0.00	−0.04; 0.04	0.00	0.979
Age	0.16	0.13; 0.20	0.12	<0.001	0.12	0.08; 0.16	0.08	<0.001

In line with H1a, passive health-related SMU was weakly but significantly related to COVID-19 anxiety (*β* = 0.13, *p* < 0.001). Active health-related SMU (H1b) was also positively associated with COVID-19 anxiety (*β* = 0.24, *p* < 0.001).

Health anxiety was positively related to both passive health-related SMU (H2a; *β* = 0.27, *p* < 0.001) and active health-related SMU (H2b; *β* = 0.24, *p* < 0.001), supporting H2. We further examined the moderating effect of health anxiety on the effects of health-related SMU on COVID-19 anxiety (H3). A significant moderating effect was found for both passive (H3a; *b* = 0.08, *p* < 0.001) and active (H3b; *b* = 0.07, *p* < 0.001) health-related SMU, supporting our hypothesis.

**Figure 3:**
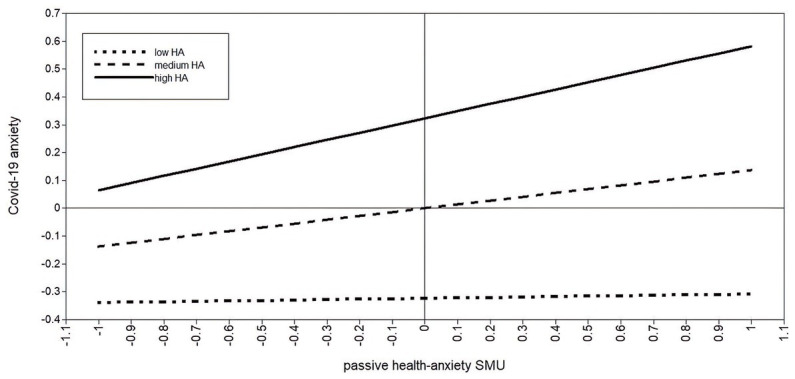
Moderating role of health anxiety between passive health-related SMU and COVID-19 anxiety.

**Figure 4:**
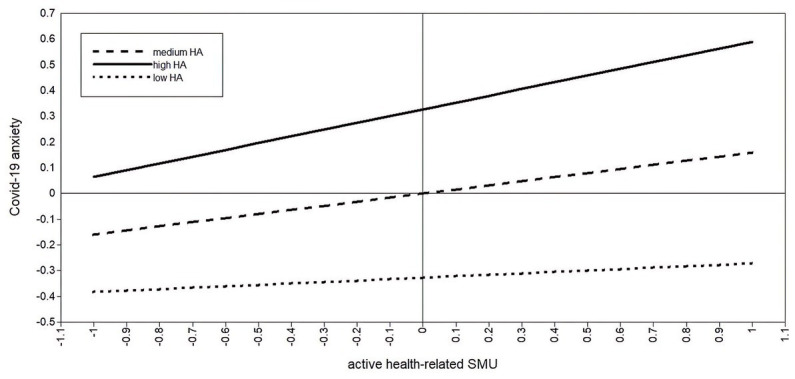
Moderating role of health anxiety between active health-related SMU and COVID-19 anxiety.

Using simple slopes analysis, we explored the moderation effect for adolescents with low (−1 SD), medium, and high (+1 SD) health anxiety (see Table 4; Figures 3, 4). For adolescents low in health anxiety, neither passive (*b* = 0.02, *p* = 0.623) nor active (*b* = 0.06, *p* = 0.206) health-related SMU were related to COVID-19 anxiety. For participants with medium health anxiety, both passive (*b* = 0.14, *p* < 0.001) and active (*b* = 0.16, *p* < 0.001) health-related SMU were positively associated with COVID-19 anxiety. Similar results for passive (*b* = 0.26, *p* < 0.001) and active (*b* = 0.26, *p* < 0.001) health-related SMU were found for adolescents high in health anxiety.

**Table 4:** The results of simple-slope analyses for both moderation effects.

	*b*	95CI b	*p*
Health anxiety * passive health-related SMU	0.12	0.06; 0.15	<0.001
Low health anxiety	0.02	−0.03; 0.14	0.623
Medium health anxiety	0.14	0.10; 0.22	<0.001
High health anxiety	0.26	0.19; 0.33	<0.001
Health anxiety * active health-related SMU	0.10	0.08; 0.16	<0.001
Low health anxiety	0.06	−0.05; 0.08	0.206
Medium health anxiety	0.16	0.08; 0.19	<0.001
High health anxiety	0.26	0.18; 0.33	<0.001

In line with H4, eHealth literacy was positively related to both passive health-related SMU (H4a; *β* = 0.22, *p* < 0.001) and active health-related SMU (H4b; *β* = 0.14, *p* < 0.001).

To explore H5, we tested whether experience with COVID-19 affects health-related media use. Experience with mild COVID-19 was significantly negatively related to both passive health-related SMU (H5a; *β* = −0.05, *p* < 0.013) and active health-related SMU (H5b; *β* = −0.11, *p* < 0.001). The association between experience with mild COVID-19 and passive health-related SMU was too weak to support H5a. H5b was supported as experience with COVID-19 was associated, albeit weakly, with active health-related SMU.

The experience with severe COVID-19 and health-related SMU were positively associated; however, the effect sizes for passive health-related SMU (H5c; *β* = 0.06, *p* = 0.01) and active health-related SMU (H5d; *β* = 0.05, *p* = 0.01) were too weak to support the hypotheses.

Contrary to our expectations, experience with mild COVID-19 was unrelated to COVID-19 anxiety (H6a; *β* = −0.03, *p* = 0.16). Experience with severe COVID-19 was positively associated with COVID-19 (H6b; *β* = 0.07, *p* < 0.001), but too weakly to support the hypothesis. Overall, H6 was not supported.

## 4. Discussion

The COVID-19 pandemic presents a challenge for adolescent mental health. This study examined individual factors associated with COVID-19 anxiety in adolescence. We expected health-related SMU to present such a factor and its frequency to be related to increased COVID-19 anxiety. We framed this relationship with three individual variables which we expected to be associated with health-related SMU: health anxiety, eHealth literacy, and experience with COVID-19. We expected health anxiety to also moderate the association between health-related SMU and COVID-19 anxiety. We add to the previous research conducted mainly at the beginning of the COVID-19 pandemic by presenting data collected during a later period.

### 4.1. Health-related SMU and COVID-19 anxiety

We expected health-related SMU to relate to COVID-19 anxiety positively, as shown in previous research (Bendau et al., 2021). To better understand this relationship, we newly divided media use into passive and active health-related SMU. More frequent passive health-related SMU was associated with higher COVID-19 anxiety, which is in line with previous literature (Valkenburg et al., 2022). It is likely that more frequent health-related media use increases the chance of finding distressing information, thus escalating the search and pursuing a worrisome topic and consequent rumination (Singh and Brown, 2016).

Contrarily, the literature was inconsistent on whether active health-related SMU also leads to a decrease, an increase, or no change in well-being (Thorisdottir et al., 2019; Keles et al., 2020; Soroya et al., 2021). In our study, active health-related SMU followed the same trend as the general and passive health-related SMU and was positively associated with COVID-19 anxiety. Our finding is in line with Valkenburg et al. (2022), who suggest that passive and active health-related SMU may not differ.

Such a finding could be related to the nature of social media, where more active use does not necessarily mean more control over one’s use, as suggested by Soroya et al. (2021). On social media, more active engagement with the content can lead to more similar content (Modgil et al., 2021), which makes the use more difficult to control. Nonetheless, these findings need to be interpreted with caution. The cross-sectional nature of this study allows for the interpretation of the relationship between health-related SMU and COVID-19 anxiety in both directions and even reciprocally (te Poel et al., 2016).

### 4.2. The effect of health anxiety

In line with the DSMM, we found health anxiety to be positively associated with health-related SMU and strengthen its relationship with COVID-19 anxiety. People with higher health anxiety are typically more worried about their health and more conscious about bodily symptoms (Singh and Brown, 2016). Such concerns translate into increased health-related media use, both passive and active, such as commenting on health-related content (te Poel et al., 2016).

Our study newly shows that health anxiety strengthened the association between both kinds of health-related SMU and COVID-19 anxiety. For adolescents with mean and high health anxiety levels, both kinds of health-related SMU were positively associated with COVID-19 anxiety. For adolescents with a low level of health anxiety, there was no such relationship. This may be due to different modes of health-related SMU in the low health anxiety group compared to the more anxious ones.

Health-related topics range from disease-related information to healthy lifestyle promotion (Singh and Brown, 2014), which are not equally distressing or pandemic-related, and, therefore, could contribute to COVID-19 anxiety differently. When reading about symptoms, users with low and high health anxiety are equally likely to reach an escalation (i.e., pursue an unlikely and grave explanation for the symptoms), which then leads to increased anxiety (Singh and Brown, 2014). However, when not instructed on what to search, people with different levels of health anxiety may differ in how they use the media. Such variance may present the main difference between adolescents with low and high health anxiety. Future research could focus on such differences and on discovering kinds of health-related SMU that would be generally less disturbing than others.

### 4.3. eHealth literacy

The level of self-perceived eHealth literacy determines the engagement in media use (Wong and Cheung, 2019) and its frequency (Chang et al., 2015). Our findings also show that perceiving oneself as eHealth literate appears to be a vital factor for initiating health-related SMU and using the media more frequently. This confirms the impact of self-perceived eHealth literacy on health-related SMU, although likely different than the objective eHealth literacy (Maitz et al., 2020).

### 4.4. Experience with COVID-19 and health-related SMU

In line with Houston et al. (2018), we studied the effect of experience with mild and severe COVID-19 on health-related SMU. To the best of our knowledge, we are the first to have tested such a relationship in the pandemic context. Our findings partially support the hypotheses. The experience with mild COVID-19 was associated with decreased active health-related SMU. This may be due to some kind of active health-related SMU that is more common for people who have had no experience with mild COVID-19. However, we were unable to identify such use because we only focused on the frequency. Adolescents who have encountered mild COVID-19 may engage with posts about COVID-19 less intensively, as they can rely on their personal experience. However, future research is needed to fill this gap.

The effects of experience with mild COVID-19 on passive health-related SMU and the experience with severe COVID-19 on any SMU were marginal. This is likely due to the topics endorsed by adolescents, such as healthy lifestyle, that may not be influenced much by the experience with COVID-19, especially when undergone by someone else. That said, we studied an association with the frequency of health-related SMU, while Houston et al. (2018) suggest that the disaster position can also impact the way of media use. Research might focus on specific purposes for the health-related SMU that would differ for people with different experiences with COVID-19.

### 4.5. Experience with COVID-19 and COVID-19 anxiety

We further tested the direct association between experience with COVID-19 and COVID-19 anxiety. The experience with mild COVID-19 was positively related to COVID-19 anxiety, while experience with severe COVID-19 had the opposite effect. However, both effects were too weak. Such findings can be explained in several ways.

By the time of our data collection, the occurrence of COVID-19 became more common and likely less distressing. Also, experience with mild COVID-19 outpatients may be reassuring compared to the overall initial depictions of the disease in the media, such as severely ill patients and people unable to visit their dying relatives (Tsamakis et al., 2021). In studies from the beginning of the pandemic, any kind of experience with COVID-19 was associated with increased COVID-19 anxiety, while actual experience with COVID-19 was rare (e.g., 0.5%; Cao et al., 2020). During the second pandemic wave, when 30% of the respondents already knew someone with mild COVID-19 and 10% knew someone hospitalized or dead, no relationship between this experience and COVID-19 anxiety was found (Fruehwirth et al., 2021).

Contrarily, the experience with severe COVID-19 is in line with the distressing depictions in the media. No relationship between experience with severe COVID-19 and COVID-19 anxiety can be explained rather by our study’s timing. It is reasonable to expect that most of our respondents had had their experience with COVID-19 sometime before the data collection, most likely between October 2020 and May 2021, when the infection rates were high (MZČR, 2022). If so, then we can cautiously say that one’s experience with COVID-19 may not affect their COVID-19 anxiety in a long-term perspective. Future studies should test this finding for different points of the pandemic.

### 4.6. Contributions to the DSMM theory

This study was framed by the DSMM and its extensions. In line with Proposition 1, the health-related SMU was related to several differential susceptibility variables, including the disaster position proposed by Houston et al. (2018). We partially supported Houston et al.’s (2018) claim for the pandemic setting. In line with Houston et al. (2018), we encourage further research to focus on the way health-related SMU changes for people with different experiences with COVID-19.

The claim of Proposition 3, that differential susceptibility variables predict the media use and moderate its impact on the response states, appeared to be valid too. In the current study, health anxiety played this dual role, and it was directly associated with the SMU and strengthened its relationship with COVID-19 anxiety.

We followed the version of the DSMM developed by Dedkova et al. (2022), who point out that ICT use must be well-specified (e.g., in terms of activity or engagement). To overcome contrary findings in the literature, we assessed active and passive use separately. Our findings suggest that active and passive health-related SMU are related and work in similar ways. In contrast, to explain the difference in media effects between various users, we need to compare health-related online activities on a more differentiated level than just a dichotomy.

### 4.7. Limitations

Several limitations need to be taken into account. The cross-sectional nature of our data does not allow for causational explanations. While health anxiety is a relatively stable trait, and experience with COVID-19 is of an external origin, other variables are prone to change and may influence each other in both directions, such as health-related SMU and COVID-19 anxiety. Longitudinal studies are needed to determine the directions.

Also, we only focused on individual and pandemic-related variables as the factors associated with health-related SMU. Future research should consider other potential variables, especially the social-related ones (e.g., social support or parental support), which can influence health and COVID-19 anxiety as well as health-related SMU.

For some variables, the source of information may also present a shortcoming. Experience with COVID-19 was reported by the caregiver, which might have omitted some of the adolescents’ experiences, especially the less stressful ones. The health-related SMU was self-reported, which is a common, but potentially unreliable way to obtain media use data, as time online is often difficult to estimate (Ernala et al., 2020).

Finally, we expected experience with COVID-19 to be an essential situational factor related to COVID-19 anxiety. Nevertheless, it is possible that, at the time of the data collection, there were other potentially stressful contextual factors, such as the debate about COVID-19 vaccination, that were not considered.

## 5. Conclusion and practical implications

This study tested an association between health-related SMU and COVID-19 in light of adolescents’ levels of health anxiety, eHealth literacy, and experience with mild and severe COVID-19. For adolescents with a moderate and high level of health anxiety, both active and passive health-related SMU were positively associated with COVID-19 anxiety. For adolescents with low health anxiety, health-related SMU was not associated with COVID-19 anxiety. Consequently, cutting back on health-related SMU may help reduce or prevent COVID-19 anxiety in health-anxious adolescents, but it may not be effective for adolescents with low health anxiety. Contrarily, identifying and avoiding specific activities that present a more significant source of stress than the others could lead to safer health-related media use without losing its benefits.

Several individual variables were associated with health-related SMU. Adolescents higher in health anxiety and self-perceived eHealth literacy used social media for health-related purposes more often. Conversely, adolescents who knew someone with mild COVID-19 were less likely to actively use social media for health-related purposes. However, the experience with COVID-19 did not have a substantial impact on COVID-19 anxiety.

To the best of our knowledge, we are the first to have applied the DSMM to the area of health-related internet use and to the context of the COVID-19 pandemic. The DSMM was a useful model for formulating hypotheses about health-related SMU. Our results underline that specification of the assessed type of media use is vital. To summarize, this study contributes to both the theory and the understanding of the impact of the pandemic on adolescents’ mental health.

## Data availability statement

The raw data supporting the conclusions of this article will be made available by the authors, without undue reservation.

## Ethics statement

The studies involving human participants were reviewed and approved by the Ethical Committee of Masaryk University. Written informed consent to participate in this study was provided by the participants’ legal guardian/next of kin.

## Author contributions

AL and DS designed the study that is the source of the article. AL drafted the manuscript and designed, conducted, and wrote the results in consultation with NK. All authors contributed to the article and approved the submitted version.

## Funding

This work has received funding from the Czech Science Foundation, project no. 19-27828X.

## Conflict of interest

The authors declare that the research was conducted in the absence of any commercial or financial relationships that could be construed as a potential conflict of interest.

## Publisher’s note

All claims expressed in this article are solely those of the authors and do not necessarily represent those of their affiliated organizations, or those of the publisher, the editors and the reviewers. Any product that may be evaluated in this article, or claim that may be made by its manufacturer, is not guaranteed or endorsed by the publisher.
